# Drug loss while crushing tablets: Comparison of 24 tablet crushing devices

**DOI:** 10.1371/journal.pone.0193683

**Published:** 2018-03-01

**Authors:** Min Yew Thong, Yady J. Manrique, Kathryn J. Steadman

**Affiliations:** School of Pharmacy, The University of Queensland, Brisbane, Queensland, Australia; Waseda University, JAPAN

## Abstract

This study investigated 24 tablet crushing devices for drug loss using different methods to recover the crushed tablet. 24 devices were compared: 3 with disposable cups, 6 with disposable bags, 12 without separate vessels and 3 types of mortar and pestle. One paracetamol tablet was crushed and recovered by tapping the powder out. Where appropriate, depending on crusher size and manufacturer instructions, the powder was also recovered by mixing with water or food. Paracetamol recovery (quantity that can be delivered to a patient) and leftover (quantity remaining in the device) were measured using a validated UV method and the entire experiment was replicated 3 times. Drug recovery ranged from 86.7–98.1% when the crushed tablet was tapped out of the crushers (average loss 5.8%). Significant losses were measured for 18 crushers, particularly manually operated hand-twist crushers with a serrated crushing surface, and some devices with disposable bags or cups. Rinsing the crushed powder with water once resulted in an average of 24.2% drug loss, and this was reduced to 4.2% after a second rinse. If crushing is unavoidable, maximizing medication delivery to the patient is essential. Rinsing twice resulted in similar paracetamol recovery to tapping the powder out; however only water rinses have the potential for direct consumption by the patient, minimizing drug loss across the entire crushing and transfer process.

## Introduction

Tablets are often crushed to facilitate easier medication administration. Tablet crushing should be approached with caution because it can alter the pharmacokinetic properties, therapeutic efficacy and safety of the medication. When managing patients unable to swallow solid oral dosage forms, substitution with an alternative oral formulation (e.g. liquid, orodispersible, effervescent), or alternative route of administration is more appropriate. If no alternative is available, crushing the solid oral dosage form may be considered and most guidelines recommend the use of mortar and pestle or a pill crusher [1–4]. As there is no standard protocol available, crushed medications are then delivered to the patient by mixing with a food vehicle such as water, juice, jam, yoghurt, honey, applesauce or thickened fluid [1,5–7] or for patients undergoing enteral intubation the powder is suspended in water and flushed through the tubing [8].

Drug loss is frequently observed when tablets are crushed for medication administration, with powder being spilled or left behind in the vessel [6,8–10]. Lower serum drug concentrations reported for patients receiving crushed medications via nasogastric feeding tubes [11–13] may be associated with drug sorption to the plastic tubing, but observed losses during crushing, mixing and transfer may have greater impact [8]. Additionally, in aged care and hospital environments, it is common to share the crushing device among residents without cleaning between uses [1,6,8–10,14,15]. For these reasons, a range of sophisticated tablet crushing devices are available. There are two main types of manual tablet crushers; one involves lifting a lever up and down to exert a crushing pressure on the crushing pad whereas the other involves a twisting action i.e. inserting a crushing member into a container for a rotating or grinding movement to crush the tablet within the container. To reduce the potential for cross-contamination without cleaning, individual medications can be crushed within disposable vessels in the form of cups or bags. Electronic devices are also available, which operate on the same principles but have a button-activated crush to reduce user fatigue.

Despite an array of tablet crushing devices being commercially available, consideration of crusher efficiency in aiding accurate medication delivery has received only occasional attention, primarily from the perspective of medication administration for patients with enteral intubation. Measurement of powder weight was used to investigate six tablets and capsules using a mortar and pestle in the laboratory [16], and for comparison of the powder remaining after the use of a mortar and pestle or a twist-action pill crusher in a clinical setting [15]. Light transmittance through tablet particles suspended in water was used to assess powder yield for three different tablets that were crushed and resuspended in a porcelain mortar and pestle or twist-action pill crusher [17]. In these studies, loss of tablet weight has been assumed to correlate with loss of active drug, however active drug may not be homogeneously distributed through the tablet, and it is possible that excipients and active ingredients differ in their relative propensity to stick to the walls of a plastic/glass/metal crushing device, so it is expected that direct measurement of loss of active drug is more accurate and more clinically relevant than the loss of tablet weight. The drug concentration recovered at each stage of crushing, transfer, suspension in water and delivery through a tube has been measured by HPLC-UV for amiodarone crushed in an electronic grinder [13] and ticagrelor crushed with a mortar and pestle [18].

These publications show the potential for significant loss of tablet powder during crushing and transfer to the patient. Crushing with a mortar and pestle and transfer was associated with 5.5–13.3% loss of tablet weight [16] and up to 17% of amiodarone content [13]. In contrast, loss of tablet weight was between 0.0 and 4.8% following crushing and delivery to 100 patients in a hospital but the average was very low (0.63%) and difference was insignificant between mortar and pestle (0.7%) and twist-action pill crusher (0.6%) [15]. However, the loss of tablet weight was measured by scraping powder remaining in the device with a spatula and brush onto weighing paper [15]. As more powder may be removed if water is used to rinse the receptacle [16], it is likely that drug loss was underestimated by simply brushing out the remaining powder. Indeed, drug loss was low, 0.5–0.8%, when crushed ticagrelor was rinsed from a glass mortar and pestle twice with 100 mL of water into a dosing cup for oral delivery [18].

This study was designed to investigate drug recovery and loss after tablet crushing in a wide range of commercially available crushing devices, each varying in their mechanism of crushing and inclusion of disposable vessel. Additionally, as rinsing with water has been shown to be a useful way to improve recovery, this study compared different methods for recovering the powdered tablet i.e. by tapping the powder out, rinsing with water or mixing with food.

## Materials and methods

### Study design

A total of 24 tablet crushing devices tested in this study (S1 Table, S1 Fig): 3 with disposable cups, 6 with disposable bags, 12 without separate vessels and 3 types of mortar and pestle. These were selected after internet searches using the terms ‘tablet crusher’ and ‘pill crusher’ to represent the types of crushers available for purchase in Australia, UK, USA and Canada, and one of each was purchased in Australia or imported to Australia for testing. White, round and scored immediate release 500 mg paracetamol tablets (Panamax, Sanofi Aventis Australia, Macquarie Park, NSW) with hardness 99.6 ± 10.7 N (mean ± SD of 10 tablets) were tested in this study. Paracetamol is the most commonly crushed tablet in hospitals [6,7], most commonly cited as being difficult to swallow [19], and was chosen as the case study medicinal product for comparing tablet splitting devices [20]. One paracetamol tablet was weighed and then crushed. Three different methods were used to recover the crushed tablet: all 24 crushing devices were tested by tapping the powder out, 12 devices were rinsed with water, and three different foods were mixed with the powder for one device based on manufacturer recommendations. Paracetamol loss from crushing was calculated in two different ways: the weight of powder recovered using a balance, and by measuring the quantity of paracetamol recovered using UV spectroscopy based on a pharmacopoeial method [21]. Paracetamol recovery (the quantity that could be taken by the patient) and leftover (the quantity remaining in the device) were measured, and percentage of paracetamol loss (the quantity that was not recovered) was calculated for each crushed tablet. The entire experiment was conducted by the same operator and replicated three times. The methods are also available at http://dx.doi.org/10.17504/protocols.io.m9tc96n.

### Paracetamol quantification method validation

Standard solutions were prepared in the range 1–12 mcg/mL. This was prepared from a stock solution (1000 mcg/mL) containing 500 mg of paracetamol USP powder (PCCA, Matraville, NSW) in 500 mL of 0.02 M NaOH, dissolved by 15 min sonication and mixed well, and a small volume filtered through 0.45 μm nylon filter (Grace Davison Discovery Sciences, Archerfield, QLD). Aliquots of filtered stock in the range 0.1 to 1.2 mL were diluted to 100 mL with 0.01 M NaOH to prepare standard solutions with concentration of 1, 2, 4, 5, 6, 8, 10 and 12 mcg/mL at pH12 [21].

Sample solutions were prepared by taking the quantity of powder equivalent to 500 mg of paracetamol from 20 accurately weighed and powdered paracetamol tablets, and preparing the stock and dilutions as described for the standard solutions. Absorbance at 255 nm was measured on a Cary 50 Bio UV-visible Spectrophotometer (Agilent Technologies, Mulgrave, VIC). Water was used as the blank; UV absorbance of 0.01 M NaOH was no different to water.

The method was validated for specificity, linearity, sensitivity (detection limit and quantitation limit), precision (repeatability and intermediate precision) and accuracy according to International Conference on Harmonization guidelines [22]. Specificity was assessed by comparing the UV scan of 10 mcg/mL of standard and sample solutions across range of 200–400 nm, to verify the absence of interference from tablet excipients and to establish wavelength of maximum absorption, λmax, for use in paracetamol quantitation (255 nm). The eight point calibration curve constructed with standard solutions in the range 1–12 mcg/mL was prepared in triplicate and linearity was evaluated by linear regression analysis by the least square regression method. Sensitivity was evaluated by calculating the detection limit, DL = 3.3σ/S, and quantitation limit, QL = 10σ/S, using the standard deviation (σ) and slope (S) of the calibration curve. Precision was studied with respect to repeatability (intra-day) and intermediate precision (inter-day) by calculating standard deviation and % relative standard deviation (% RSD) around the mean of triplicate measurements taken on the same day (repeatability) or two consecutive days (intermediate precision) for both standard and sample solutions at concentrations of 1, 5 and 10 mcg/mL. Accuracy was assessed by conducting recovery tests at 80%, 100% and 120% of test concentration in triplicate. A quantity of paracetamol standard powder and finely powdered paracetamol tablets equivalent to 400 mg, 500 mg and 600 mg of paracetamol were transferred into a 500 mL volumetric flask, diluted and measured.

### Data collection

#### Control

Average tablet weight was calculated from the individual weights of ten paracetamol tablets. One paracetamol tablet was transferred to a 500 mL volumetric flask. 100 mL 0.1 M NaOH was added to the flask and it was made up to volume with water. The paracetamol tablet was dissolved by sonication for 15 min, and a small volume was filtered through 0.45 μm nylon filter. Then 1 mL of filtrate was diluted to 100 mL in 0.01M NaOH and the absorbance measured at 255 nm. This was replicated for all 10 paracetamol tablets.

#### Tablet crushing

Tablet crushing devices with disposable vessels (cups and bags) were used with the specific vessel supplied with or designed for use with the device (S2 Table, S2 Fig). Two empty cups (or a bag / a hand-twisted device / a syringe / mortar and pestle) were weighed, W1. One tablet was placed between the top and bottom cups (in the bag / hand-twisted device / syringe / mortar and pestle) and the weight was measured, W2. The tablet was crushed according to manufacturer instructions (where they existed). For crushing devices operated by lifting a lever up and down, the lever was lifted five times while rotating the position of cups or bags. For hand-twisted devices, the device was rotated three times by loosening and tightening the lid. For the three electronic crushing devices tested, standard grind was selected for the First Crush, and the button was pressed for one minute for the Vitacarry and 3 to 5 seconds for the Powdercrush. For the mortars and pestles, and ball and socket, the tablet was crushed for 60 seconds. The weight of the cups (or bag / device / syringe / mortar and pestle) with crushed tablet was measured, W3.

#### Powder retrieval by tapping out

The top cup (lid of hand-twisted device) was tapped to release the powder adhered to the bottom surface of the top cup (lid of hand-twisted device). The crushed tablet was poured or tapped out of the bottom cup (bag / bottom unit of hand-twisted device / syringe / mortar). The weight, W4, of the two cups (bag / device / syringe / mortar and pestle) was measured. The loss of tablet weight was calculated as weight of the individual tablet (W2-W1) minus the weight of crushed tablet recovered from tapping (W3-W4).

The crushed tablet was tapped out into a 500 mL volumetric flask via a funnel and prepared and measured as described for the control; this was assumed to be the recovered amount that could potentially be consumed by the patient. The powder that adhered to the bottom surface of the top cup (lid of hand-twisted device) and the powder remaining in the bottom cup (in the bag / in the bottom unit of hand-twisted device / in the syringe / on mortar and pestle) was transferred by exhaustively rinsing with water into a 100 mL volumetric flask. 20 mL 0.1 N NaOH was added to the flask and thereafter it was prepared and measured as described for the control. This was assumed to be the leftover that could not be taken by the patient.

#### Powder retrieval by rinsing with water

Twelve crushing devices were selected either because rinsing with water is recommended by the manufacturer or the device/vessel has the potential to be used in this way. After crushing, approximately 30 mL of water was added to the crushed tablet in the bottom cup (bag / bottom unit of the device / withdrawn into the syringe), slight agitation given and the solution was poured out immediately into a 500 mL volumetric flask as the first rinse. This rinsing process was repeated with another 30 mL of water and poured into a separate 500 mL flask to study the amount of paracetamol in the second rinse. Both solutions were prepared and measured as described for the control. The leftover powder remaining in the top and bottom cups (bag / lid and bottom unit of hand-twisted device / syringe) was transferred by exhaustively rinsing with water into a 100 mL volumetric flask. 20 mL 0.1 N NaOH was added to the flask and thereafter it was prepared and measured as described for the control.

#### Powder retrieval by mixing with food

Only the First Crush Automated Pill Crusher Gen 2 was tested with food because it is the only crushing device that has a specific manufacturer recommendation to mix the crushed tablet with food in the cups for direct delivery to the patient. After crushing, 15 g of apple sauce (Threes Three, Lidcombe, NSW), honey (Capilano, Inala, QLD) or vanilla yoghurt (Yoplait, Melbourne, VIC) was mixed with the crushed tablet in the bottom cup with a spoon. The food and powder mixture was transferred by scraping out with the spoon into a 500 mL volumetric flask via a funnel, and then prepared and diluted as described for the control. Leftover food and powder in the bottom cup and on the bottom surface of the top cup was transferred to a 100 mL flask and prepared and diluted as described for the leftover from tapping out. The absorbance measured for crushed paracetamol tablet mixed in foods was taken against a blank without crushed tablet that was otherwise prepared and diluted in the same way.

#### Data analysis

The amount (mg) of paracetamol recovered and leftover were calculated using the calibration curve. The theoretical quantity of paracetamol contained in each tablet was calculated as actual tablet weight / average tablet weight x 500 mg. The quantities of paracetamol recovered and leftover were converted into % recovery and % leftover by dividing the quantity recovered or leftover by the theoretical quantity of paracetamol in the tablet x 100. The % loss was calculated as 100 –% recovery. All results are given as mean ± sample standard deviation (SD).

The % recovery from tapping out was compared with the control (whole tablet) using one-way ANOVA followed by a Dunnett’s post hoc test. Additionally, since the porcelain mortar & pestle is a widely used low cost device that is likely to be similar to that used in other studies [15,16,18], % recovery by tapping out from the other crushing devices was compared against the porcelain mortar & pestle using one-way ANOVA and Dunnett’s post-hoc test. Paracetamol recovery by one rinse with water and two rinses with water were investigated for differences with two-way ANOVA followed by a Fisher’s LSD. Comparison of recovery using two rinses with tapping out was made using two-way ANOVA and Fisher’s LSD. Paracetamol recovery using the First Crush Gen 2 device and tapping out, two rinses with water, or mixing with honey, apple sauce or yoghurt was compared using a one-way ANOVA with Tukey multiple comparisons test of the means. All statistical analyses were performed using GraphPad Prism version 7.00 (GraphPad software, San Diego, CA), and p<0.05 was considered statistically significant.

## Results

### Method validation

The eight point calibration curve constructed in the range of 1–12 mcg/mL was linear with high correlation coefficient, 0.9999, and with limit of detection of 0.13 mc/mL and limit of quantitation 0.40 mcg/mL. For both standard and sample tests, % RSD for inter- and intra-day precision were well below 2% and recovery was over 99%.

### Powder retrieval by tapping out

The control (whole tablets treated in the same way as powder collected from the tablet crushers) showed that 99.8% of the paracetamol that is predicted to be present in each tablet could be measured using the method applied in this study. Between 86.3 and 98.1% of the paracetamol was recovered when the powder was tapped out of the devices (Table 1), which meant that between 1.9 and 13.7% of drug was lost (average 5.8%). Most of this loss was measured as being leftover in the device or vessel, and consequently there was a strong linear relationship between the % leftover and % loss (R^2^ = 0.933). Encouragingly, for six of the devices the quantity of drug recovered from crushed tablets was not statistically different to the quantity of drug recovered from whole tablets (Table 1).

**Table 1:** Percentage of paracetamol recovery, leftover and loss for one 500 mg paracetamol tablet crushed in each of 24 crushing devices (n = 3) followed by tapping the powder out. A significant difference in paracetamol recovery for each crusher compared with the control (whole tablet, n = 10) is indicated (* p < 0.05, ** p<0.01, *** p<0.001,—no difference). See Supplementary information for details and images of each crusher and disposable vessel.

	Recovery^a^ (%)			Leftover^b^ (%)		Loss^c^ (%)	
	Mean	S.D.		Mean	S.D.	Mean	S.D.
**Control**	99.81	0.57					
Crushers with disposable vessels							
With disposable cups							
**First Crush Automated Gen2**	91.02	1.67	***	7.61	1.38	8.98	1.67
**Ocelco Plastic Pillcrusher**	94.96	2.54	**	2.20	0.89	5.04	2.54
**Rhino Crush**	97.34	1.75	-	2.26	1.08	2.66	1.75
With disposable bags							
**Metal handheld**	97.87	0.41	-	0.64	0.22	2.13	0.41
**MiniTwist**	95.96	0.62	*	3.06	0.69	4.04	0.62
**Powdercrush**	94.04	2.30	***	5.56	2.17	5.96	2.30
**Roc N Crush**	92.38	3.32	***	7.32	2.18	7.62	3.32
**Quiet Crusher**	90.27	4.59	***	8.64	4.45	9.73	4.59
**Silent Knight**	86.69	3.42	***	12.43	2.82	13.31	3.42
Crushers without disposable vessels							
By hand-twisting							
*Serrated flat surface*:							
**Ultra Fine Cut N Crush**	94.65	0.82	**	3.69	0.64	5.35	0.82
**Crusher with storage**	92.74	0.90	***	6.13	1.21	7.26	0.90
**Cut/crush cups**	92.26	2.31	***	6.64	2.09	7.74	2.31
Crushing syringe*	86.31	2.15	***	9.78	1.04	13.69	2.15
*Smooth conical surface*:							
**Ergo-grip crusher**	94.95	1.58	**	4.45	0.92	5.05	1.58
**Apex Ultra tri-grip crusher**	94.24	2.54	***	5.10	1.70	5.76	2.54
*Smooth flat surface*:							
**Sabi Crush**	96.22	0.62	-	3.22	0.47	3.78	0.62
**Deluxe crusher**	95.54	0.86	*	3.17	0.58	4.46	0.86
*Smooth ball-shaped surface*:							
**Combination crusher/cutter**	97.10	1.01	-	2.14	0.43	2.90	1.01
**Crushy crusher & splitter**	95.80	1.38	*	3.28	0.95	4.20	1.38
Mortar and pestle-like							
**Ball and socket tablet pulverizer**	98.11	1.33	-	0.82	0.07	1.89	1.33
**Agate mortar & pestle**	97.74	0.93	-	0.78	0.37	2.26	0.93
**Porcelain mortar & pestle**	95.92	1.32	*	2.86	1.46	4.08	1.32
**Glass mortar & pestle**	95.29	0.86	**	3.63	0.58	4.71	0.86
Blade							
**Vitacarry automatic pill grinder**	93.60	1.20	***	3.92	0.23	6.40	1.20

* The syringe is designed for use with water, not tapping the dry powder out, and is included here for comparison only.

^a^ Paracetamol recovered from the device by tapping out: % Recovery = quantity recovered / theoretical quantity present in the tablet x 100

^b^ Paracetamol remaining in the device or disposable vessel after tapping out: % Leftover = quantity leftover in the device / theoretical quantity present in the tablet x 100.

^c^ Paracetamol that was not recovered: % Loss = 100 –% Recovery

For crushing devices with disposable bags, between 2.1 and 13.3% (average 6.6%) of drug was lost. Each device was supplied with a specific bag, five of which were 6 or 7 mil (0.15–0.18 mm) thickness polyethylene bags (see Supplementary S2 Table); these became dented during crushing and powder became trapped onto the rough surface when trying to tap it out. This was most evident for the Silent Knight and Quiet Crusher, with 12.4% and 8.6% of drug respectively being measured as leftover in the disposable bag. The resealable bag supplied with the Roc N Crush trapped powder at the seal, resulting in 7.6% drug loss. The Easy Empty Crusher Bag used with the MiniTwist device is funnel shaped and requires the perforated base to be torn or cut off to allow powder to flow out, however a small quantity of powder remained in the discarded tip. The metal handheld crusher was the only device with a disposable plastic bag not to be associated with significant drug loss when used to crush a paracetamol tablet (Table 1); the bags were 2 mil (0.05 mm) thickness plastic and without any seal.

Among the tablet crushers with disposable cups, the First Crush automated pill crusher (the most expensive of the crushers that we tested) exhibited the greatest drug loss (9%). Despite having an antistatic coating on the purpose-designed plastic cups, 7.6% of drug remained in the cups after tapping out and this was mainly due to powder that was not released from the bottom surface of the top cup on tapping. The Rhino Crush, supplied with Solo brand translucent ½ oz polystyrene soufflé cups, was associated with minimal drug loss when the powder was tapped out (Table 1).

Crushing devices without a separate disposable vessel resulted in an average of 4.7% (1.9–7.7%) drug loss with 3.6% (0.8–6.6%) left in the devices. This excludes the crushing syringe, which is not designed for powder to be tapped out and was included for comparison only. The hand-twist devices have a receptacle that contains the tablets, then a crushing member is inserted and rotated by the threaded connection to crush the tablets within the receptacle. Despite the similar crushing mechanism, they have different types of crushing surfaces. Those with a serrated surface, designed to provide a fine grind, were found to trap powder between the teeth leading to significant drug loss (5.35–7.74%). Smooth internal surfaces were associated with a range of losses that extended to low levels (2.9–5.76%).

Although the agate mortar and pestle produced very low drug loss (2.3%) when the powder was tapped out, porcelain or glass (4.1–4.7% loss) is expected to be more commonly used than agate due to lower cost (Table 1). The porcelain mortar and pestle, as the industry standard, had significantly lower drug loss (P<0.05) than the Silent Knight, Quiet Crusher, First Crush and crushing syringe when the powder was tapped out.

Powder loss to the environment was responsible for differences between the measured drug leftover and the calculated drug loss. The difference was greatest for the Ocelco Plastic Pillcrusher as 5% of drug was lost with only 2.2% remaining in the ¾ ounce paper soufflé cups, and a puff of powder was clearly visible during crushing for these paracetamol tablets. The Vitacarry automatic pill grinder had a difference of 2.5% between loss and leftover because it was very difficult to remove all of the powder from the grinding mechanism for measurement of the leftover drug. The difference was negligible for the Roc N Crush resealable bags (0.3%), and Powdercrush bags for which manufacturer instructions specified folding the top (0.4%), as the contents were unable to escape during crushing. The mortar and pestle is the most obvious open crushing environment, but for these paracetamol tablets the mortar and pestle showed a difference between drug loss and leftover of only 1.1 to 1.5%, which was no different to the average difference for all crushing devices (1.1%).

**Fig 1:**
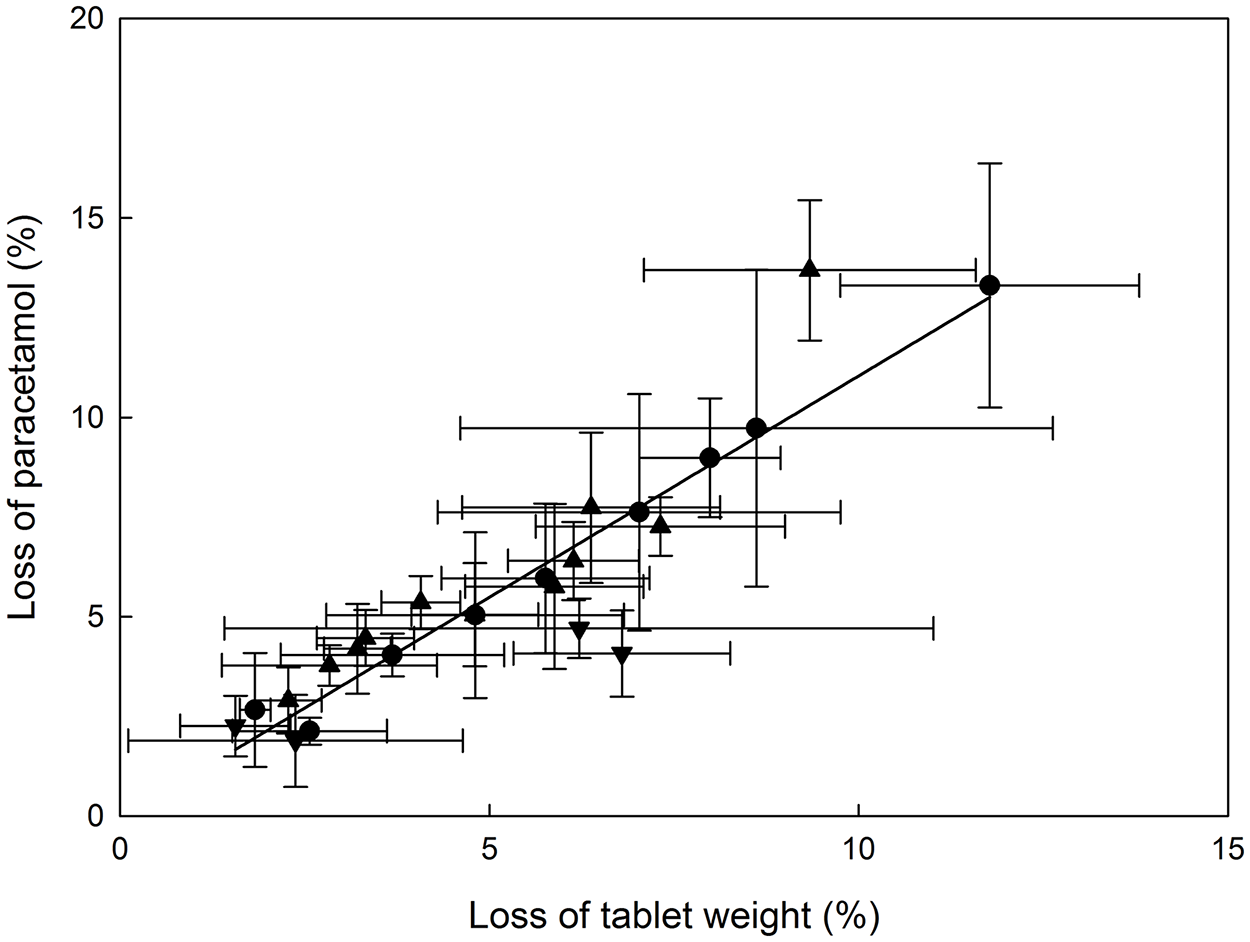
Co-plot of the two approaches to quantifying drug loss during crushing. Drug concentration was measured by UV (% paracetamol loss = 100 –% recovered) and tablet weight was measured using a balance (% tablet weight loss = weight of crushed tablet recovered / weight of whole tablet x 100). The symbols are the mean measurements derived from powder tapped out of 24 tablet crushers that were used to crush one 500 mg paracetamol tablet; crushers with disposable vessel (●), without disposable vessel (▲), mortar and pestle-like (▼). The linear regression through the means (y = 1.110x−0.064) has R^2^ = 0.852.

This experiment focused on the concentration of paracetamol recovered rather than the weight of crushed tablet recovered, but these two approaches to assessing tablet loss during crushing were closely related, with a linear regression accounting for 83.5% of the variance in the means (Fig 1). The porcelain mortar and pestle deviated the most below the regression line, indicating less paracetamol was lost than expected from the powder weight loss, and the crushing syringe deviated the most above the line.

### Powder retrieval by rinsing with water

**Fig 2:**
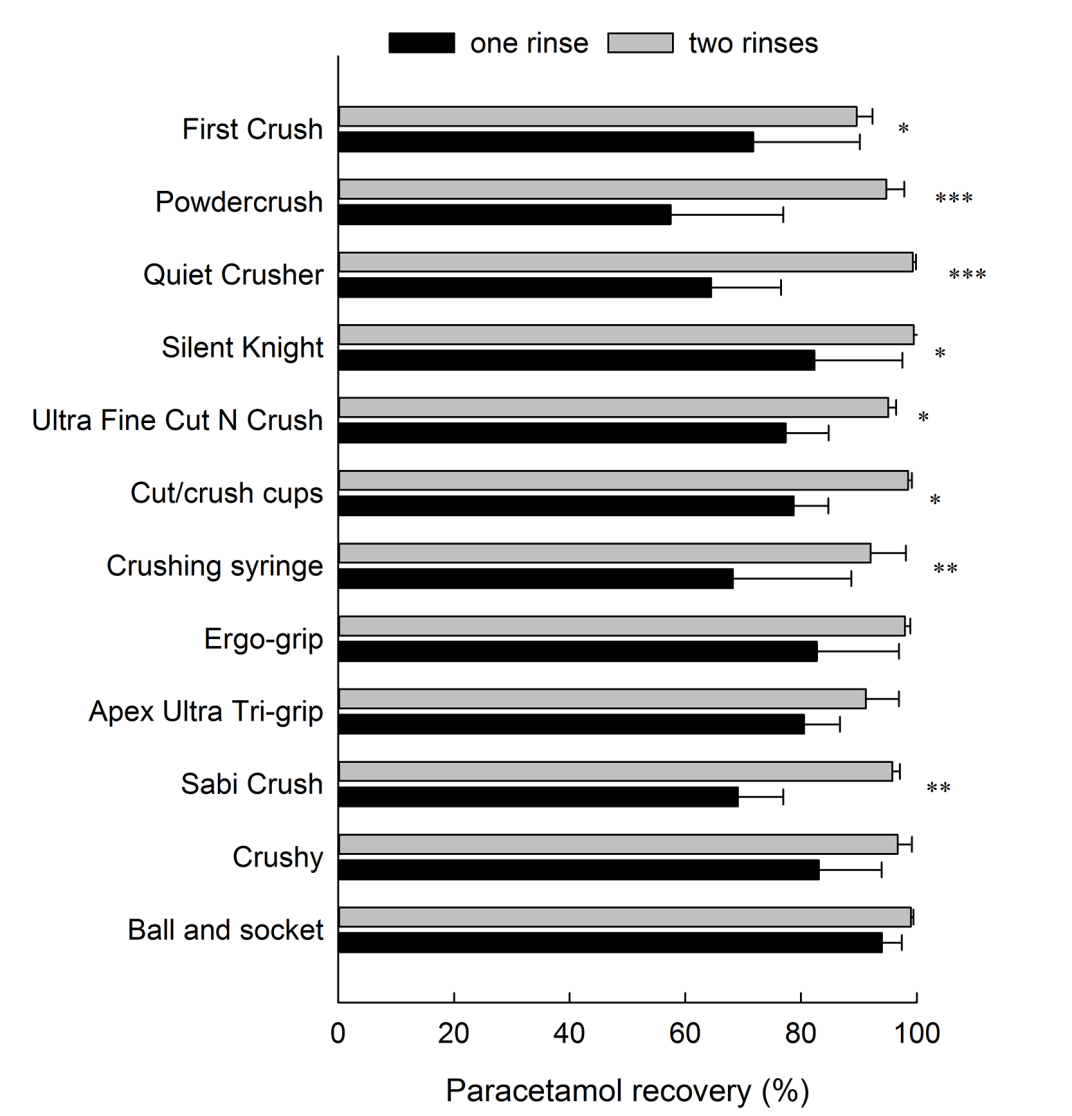
Comparison of one and two rinses with water on recovery (%) of a 500 mg paracetamol tablet crushed using 12 tablet crushing devices. A significant difference in recovery between one and two rinses is indicated (* p<0.05, ** p<0.01, ***p<0.001).

Of the 24 devices tested in this study, 12 had a vessel that had the potential to be used as a drinking cup. Using one rinse of water recovered as little as 57.5% through to 94% of the dose depending on the crusher (Fig 2). This is an average loss of 24.2%, ranging from 6% with the ball and socket up to 42.5% for the Powdercrush. Two rinses with water reduced drug loss to an average of 4.2% (0.5–10.4%), which was a significant improvement in comparison to one water rinse for most (8/12) of the devices (Fig 2). Two water rinses resulted in a similar recovery to tapping out for most (8/12) devices, but two rinses was a significant improvement in recovery for the pill cups, Quiet Crusher, Silent Knight and syringe. The crushing syringe is designed to have fluid drawn in and ejected out as a means to deliver the crushed tablet into a feeding tube; a single rinse with water resulted in 68.3% recovery of paracetamol, which increased to 92.0% with the second rinse.

### Powder retrieval by mixing with food

**Fig 3:**
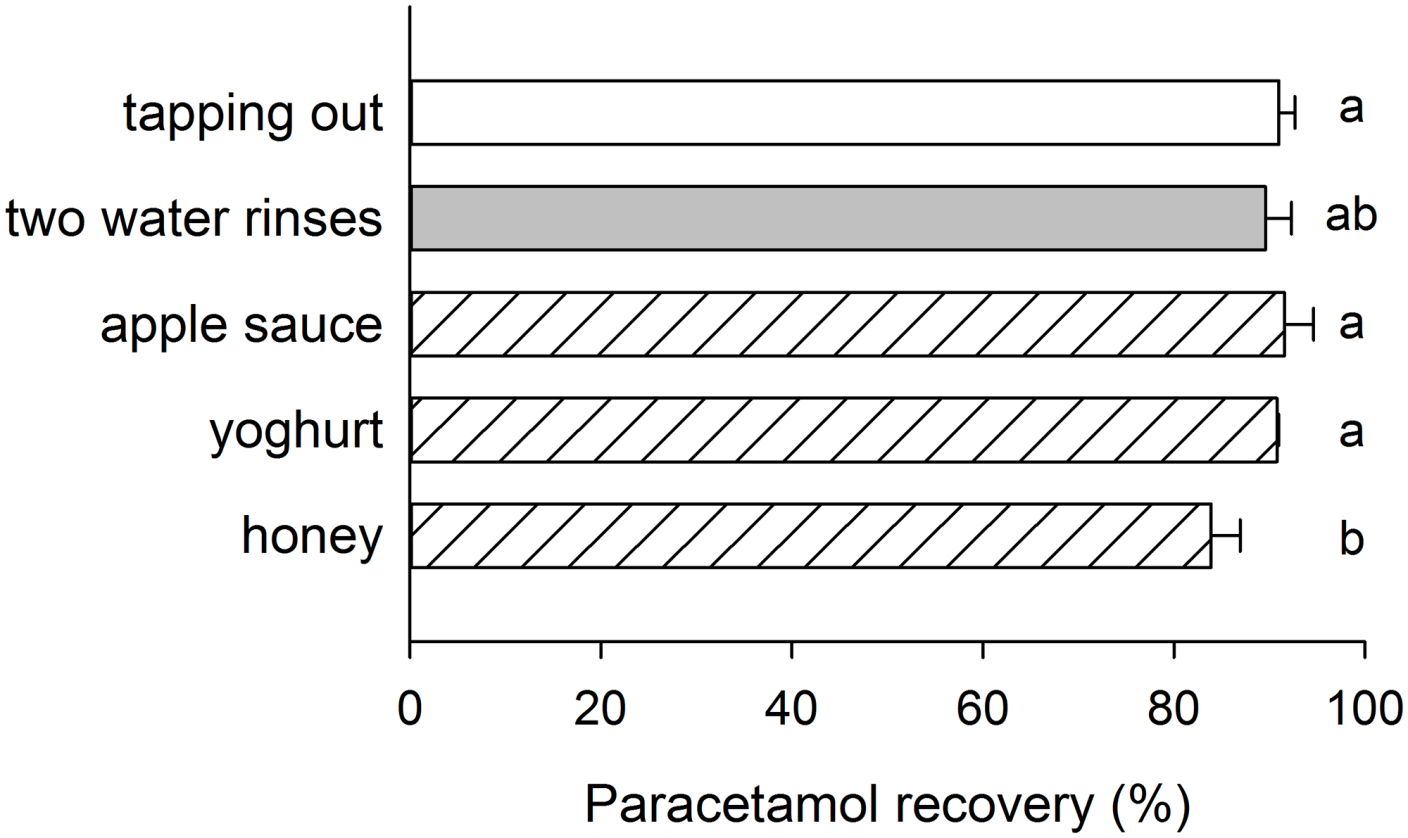
Recovery of 500 mg paracetamol tablet after crushing with the First Crush Automated Pill Crusher Gen 2 when the dry powder was tapped out of the disposable cups, rinsed with water twice, or scraped out with a spoon after mixing with apple sauce, yoghurt or honey. Bars that do not share the same lower case letter are significantly different (p<0.05).

Of the three food items tested with the First Crush, drug recovery using yoghurt (95.2%) and apple sauce (93.7%) was no different to either tapping the powder out or rinsing with water twice (Fig 3). In each case, the lost drug was measured as being leftover in the vessel primarily because the powder adhered to the lower surface of the top cup and so mixing food into the contents of the bottom cup did not help to recover this powder. Honey was particularly difficult to remove from the cup and additional leftover drug was measured in the cup for this reason, leading to a significant reduction in drug recovery to 83.9% (Fig 3).

## Discussion

To minimize drug loss, we recommend that water is added to the crushed tablet in the crushing device or disposable vessel and consumed directly from the device or vessel, with a second rinse being essential. Crushing the tablet and rinsing it with water once resulted in an average of 24% drug loss, depending on the crushing device used, but a second rinse with water reduced drug loss to 4%. If the entire portion is consumed directly from the crushing device or its disposable vessel, drug loss from rinsing with water twice can be considered to be the total loss from the whole administration process. It is expected that the percentage of recovery would increase with the number of rinses and volume of fluid used. Up to 99.5% of drug was recovered by rinsing with 100 mL of water twice and stirring repeatedly [18] but the workload burden for nurses and the ability of patients to swallow large amounts of fluid have to be taken into consideration. By recommending two consecutive rinses with water without stirring, at least 95% of active ingredients will be delivered to the patients with most (9/12) of the crushing devices tested.

As water may pose safety challenges for patients with dysphagia, crushed tablets are often mixed with naturally thick food such as jam, yoghurt or honey, or with natural gum-based thickeners to reduce the risk of aspiration [23]. Standard clinical practice in hospitals [7,15] and nursing homes [6] is to pour the powder out of the crushing device into another receptacle containing the food or thick fluid. Drug loss when the powder was tapped out ranged from 1.9 to 13.7%, with at least 95% of the paracetamol being recovered by only 11 out of the 24 devices. Manually operated hand-twist crushers with a serrated crushing surface, and some devices with disposable bags or cups, were particularly associated with significant losses. However, unless the vessel containing the food or fluid is licked clean, it is unlikely that patients consume the entire portion and so drug loss across the whole administration process will be greater than the values measured here. The additional receptacle can be removed from the process if the food or thick fluid is mixed with the crushed tablet in the crushing device or its disposable vessel. Only one of the devices tested in the present study, First Crush, had specific manufacturer instructions for use in this manner, and yoghurt or applesauce were equivalent to two rinses with water in terms of drug recovery. No other devices were tested with food because their vessels were considered to be inappropriate or too small for easy addition, mixing and consumption of the required quantity of food or thick liquid.

Drug loss from crushing in the porcelain mortar and pestle followed by tapping the powder out in our experiment (4%) is in accordance with the 5.5% of sotalol tablet weight that was lost under similar experimental conditions [16]. Our recommendation of rinsing the tablet crushers twice with water is supported by that study as loss of tablet weight was shown to be reduced significantly when water was added into the mortar for transferring [16]. Although the authors measured the loss of tablet weight but not the loss of active drug, our experiment has indicated a strong correlation between loss of powder weight and loss of active drug for paracetamol tablets (Fig 1).

The US Food and Drug Administration recommends less than 3.0% loss of mass of tablet upon subdivision [24]. If we apply this guideline to tablet crushing, our study shows that many of the tablet crushers did not meet this guideline when crushed tablet was tapped out (19/24) or rinsed with water twice (7/12). According to the British Pharmacopoeia [21], paracetamol tablet content should fall within the limits of 95–105% of the labelled amount. Almost half of the tablet crushers (11/24) would result in more than 5% loss of paracetamol after crushing and tapping the powder out, and so delivery of less than 95% of the intended dose in comparison to a whole tablet.

Apart from drug loss, there are concerns for drug interactions and safety. Crushing devices are often shared between patients, and without cleaning, in nursing homes and hospitals [1,6,9]. Even with careful crushing and transferring using porcelain mortar and pestle, our study shows that approximately 3% of drug was left in the crushing device and can potentially transfer to the next patient if not cleaned between administrations. Aerosolization during crushing and transfer may expose the carers or nurses to allergic reaction or even toxic inhalation especially when crushing chemotherapy drugs [25]. Crushing with a mortar and pestle can cause measurable drug aerosolization, and the use of closed environment crushing has been recommended, with dispersion in water within the same receptacle to minimise drug loss and aerosolization [17]. In our study, loss to the surroundings or aerosolization can be assessed by calculating the difference between drug loss and drug leftover; the mortar and pestle had a similar amount of powder escaping to the environment when compared to other crushing devices. Tapping the powder out risks having greater loss to the surroundings (average 1.1%) than mixing the powder with water (0.2%). Using a resealable bag or folding the top opening of the bag when crushing, and recovering the powder by thorough rinsing with water is recommended where it is important to reduce the possibility of aerosolization.

There are two important limitations to this study. Firstly, only one type of tablet was used, an immediate release paracetamol tablet. Considering that losses have been shown to differ between tablet types, and that an immediate release tablet has previously been shown to have the least loss of tablet weight during crushing [16], the results obtained in the present study may not be applicable to other types of tablets. Secondly, the operator was a pharmacy student without experience in preparation of medication in a clinical setting, however the process of crushing was conducted cautiously to avoid spillage so results may be conservative in comparison to real life. The efficiency of tablet crushing devices should not be evaluated by drug loss only; many other aspects may need to be considered such as ability to produce fine and uniform powder, possibility of cross contamination between users, durability, level of noise, portability, usability and cost effectiveness.

### Conclusion

Healthcare professionals and patients need to recognise that tablet crushing can result in significant drug loss. A standard protocol of at least two consecutive rinses, with the fluid being directly transferred from the crushing device or disposable vessel to patients, is recommended as best practice for all tablet crushers. We call on manufacturers to improve crusher design in order to minimise drug loss and maximise medication delivery; i.e. a device that ensures a sealed environment for crushing followed by mixing with a fluid and subsequent consumption from the same receptacle. Furthermore, as far as we are aware, there are currently no regulations or guidelines that relate specifically to tablet crushers in any country, and we believe that this urgent rectification. However, further research is required to determine whether crusher performance varies between tablet types and users, and the extent to which research laboratory handling reflects drug loss during clinical use.

## Supporting information

S1 Fig Images of the crushers tested in this study. (JPG)

S2 Fig Images of disposable vessels that were used with the crushing devices. (JPG)

S1 Table Details of each crushing device tested in this study. Approximate device costs are shown in USD excluding freight charges ($ <10, $ $ 10–30, $ $ $ 30–100, $ $ $ $ >100).
(DOCX)

S2 Table Description of disposable vessels used with crushing devices. (DOCX)
