# Supplementary figures and images for: Drug loss while crushing tablets: Comparison of 24 tablet crushing devices
Source: PLoS One. 2018 Mar 1;13(3):e0193683. doi: 10.1371/journal.pone.0193683 (PMC5832315; doi:10.1371/journal.pone.0193683)

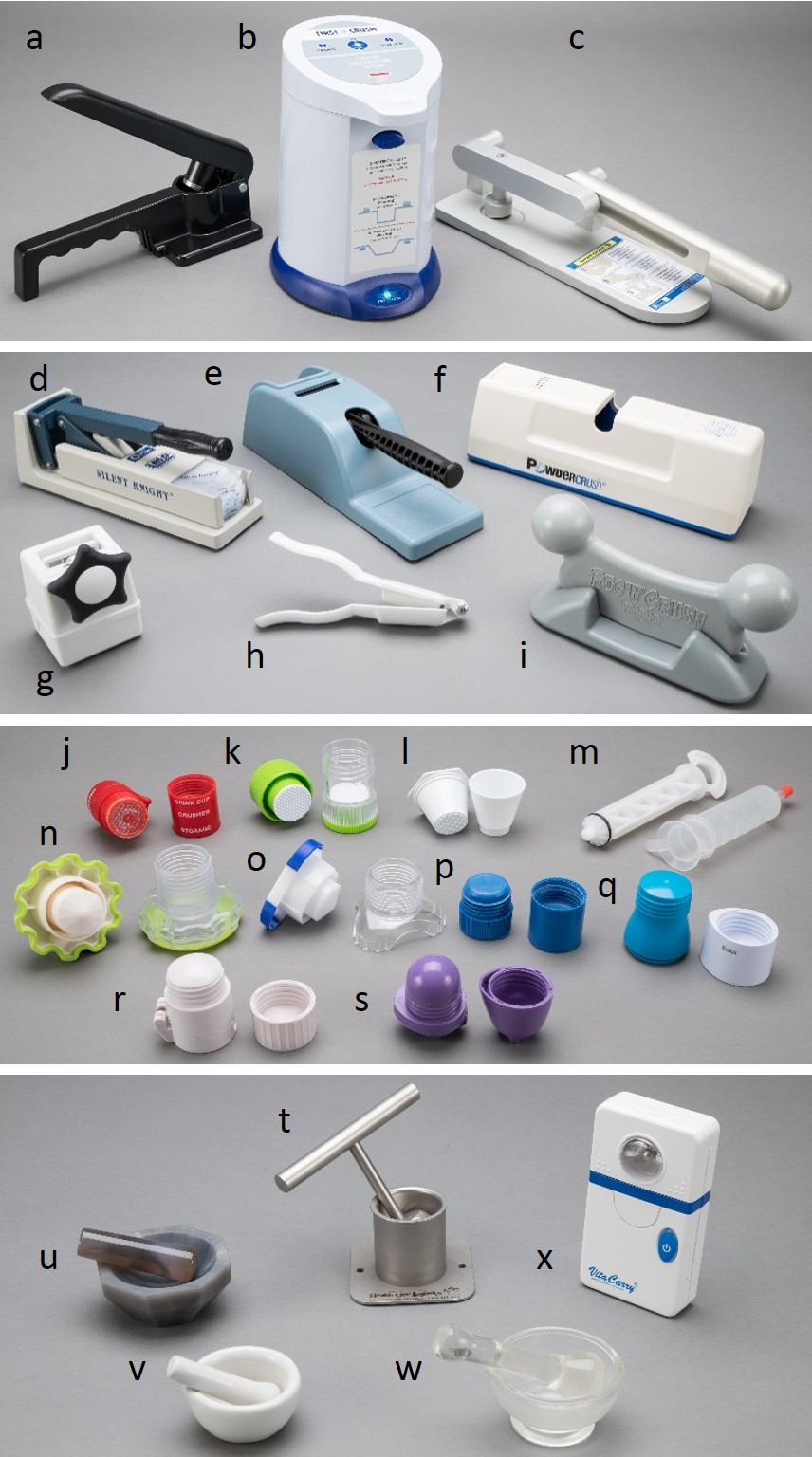

Supplement: S1 Fig — (JPG) [file pone.0193683.s001.jpg]

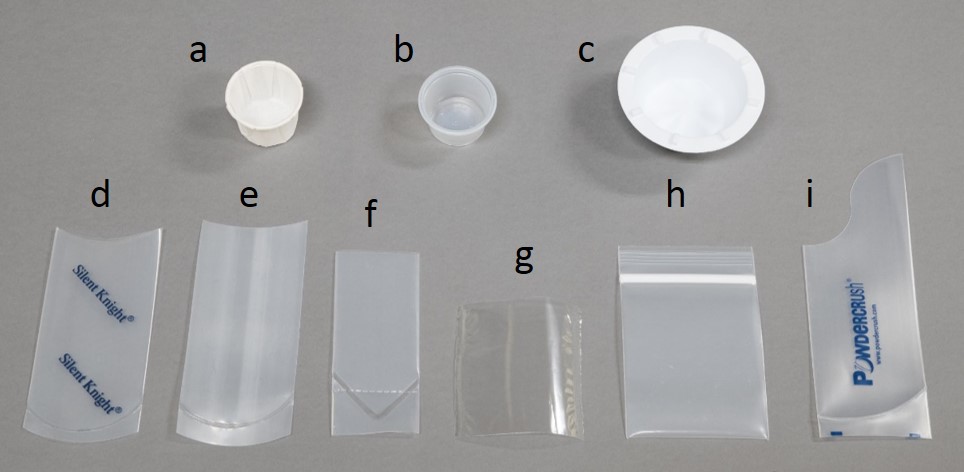

Supplement: S2 Fig — (JPG) [file pone.0193683.s002.jpg]
