# Supplementary material for: Drug loss while crushing tablets: Comparison of 24 tablet crushing devices
Source: PLoS One. 2018 Mar 1;13(3):e0193683. doi: 10.1371/journal.pone.0193683 (PMC5832315; doi:10.1371/journal.pone.0193683)
Supplement: S1 Table — Approximate device costs are shown in USD excluding freight charges ($ <10, $ $ 10–30, $ $ $ 30–100, $ $ $ $ >100). (DOCX) [file pone.0193683.s003.docx]

**S1** **Table**

| Crushers with disposable vessels | | Mode of action* | Manufacturer / Supplier | Item in Fig. S1 |
| --- | --- | --- | --- | --- |
| With disposable cups |  |  |  |  |
| **First Crush Automated Pill Crusher Gen2** | $$$$ | Electronic,  twist | First Wave Products Group  Batavia NY, USA  firstwaveproducts.com | b |
| **Ocelco Plastic Pillcrusher** | $$ | Lever press | Ocelco  Brainerd, MN, USA  pillcrusher.com | a |
| **Rhino Crush Tablet Crusher** | $$$$ | Lever press | Webstercare  Leichhardt NSW, Australia  webstercare.com.au | c |
| With disposable bags |  |  |  |  |
| **Metal handheld pill crusher** | $$ | Lever press | Ocelco  Brainerd, MN, USA  pillcrusher.com | h |
| **MiniTwist Quiet Crusher** | $$ | Twist | Health Care Logistics Inc.  Circleville, OH  gohcl.com | g |
| **Powdercrush** | $$$$ | Electronic,  press | Manrex Ltd  Winnipeg, MB, Canada  powdercrush.com | f |
| **Roc N Crush** | $$$ | press | Health Care Logistics Inc.  Circleville, OH  gohcl.com | i |
| **Quiet Crusher** | $$$ | Lever press | Health Care Logistics Inc.  Circleville, OH  gohcl.com | e |
| **Silent Knight Pill Crusher** | $$$ | Lever press | Links Medical Products Inc.  Irvine, CA, USA  linksmed.com | d |
| Crushers without disposable vessels | |  |  |  |
| By hand-twisting |  |  |  |  |
| *Serrated flat surface:* |  |  |  |  |
| **Ultra Fine Cut N Crush** | $$ | Twist,  internal screw | Apothecary Products Inc.  Minneapolis, MN, USA  apothecaryproducts.com | j |
| **Crusher with storage** | $ | Twist,  external screw | Apothecary Products Inc.  Minneapolis, MN, USA  apothecaryproducts.com | k |
| **Cut/crush cups** | $ | Twist | Health Care Logistics Inc.  Circleville, OH  gohcl.com | l |
| **Crushing syringe 60 mL*** | $ | Twist | Health Care Logistics Inc.  Circleville, OH  gohcl.com | m |
| *Smooth conical surface:* |  |  |  |  |
| **Ergo-grip tablet crusher** | $ | Twist,  external screw | Apothecary Products Inc.  Minneapolis, MN, USA  apothecaryproducts.com | n |
| **Apex Ultra tri-grip pill crusher** | $ | Twist,  external screw | Carex Health Brands  Sioux Falls, SD, USA  carex.com | o |
| *Smooth flat surface:* |  |  |  |  |
| **Sabi Crush easy pill smasher** | $$ | Twist,  internal screw | Sabi Inc.  Palo Alto, CA, USA  Sabi.com | q |
| **Deluxe tablet crusher** | $ | Twist,  internal screw | Apothecary Products Inc.  Minneapolis, MN, USA  apothecaryproducts.com | p |
| *Smooth rounded surface:* |  |  |  |  |
| **Combination crusher/cutter** | $ | Twist,  internal screw | Health Care Logistics Inc.  Circleville, OH  gohcl.com | r |
| **Crushy pill crusher & splitter** | $ | Twist,  internal screw | Francehopital  Erstein, France  francehopital.com | s |
| Mortar and pestle-like |  |  |  |  |
| **Ball and socket stainless steel tablet pulverizer** | $$$$ | press | Health Care Logistics Inc.  Circleville, OH  gohcl.com | t |
| **Agate mortar & pestle**  34 H x 75 OD (mm) | $$$ | press | # | u |
| **Porcelain mortar & pestle**  32 H x 60 OD (mm) | $ | press | # | v |
| **Glass mortar & pestle**  60 H x 70 OD (mm) | $$ | press | # | w |
| Blade |  |  |  |  |
| **Vitacarry automatic pill grinder** | $$$ | Electronic,  blade | eNNOVEA LLC  Columbus, OH, USA  ennovealife.com | x |

# mortar and pestles owned by the School of Pharmacy for many years, source could not be determined.
