# Supplementary material for: Drug loss while crushing tablets: Comparison of 24 tablet crushing devices
Source: PLoS One. 2018 Mar 1;13(3):e0193683. doi: 10.1371/journal.pone.0193683 (PMC5832315; doi:10.1371/journal.pone.0193683)
Supplement: S2 Table — (DOCX) [file pone.0193683.s004.docx]

**S2 Table.**

| Crushers with disposable vessel |  | Vessel | | Item in Fig. S2 |
| --- | --- | --- | --- | --- |
|  |  | Name | Dimension (mm) |  |
| With disposable cup |  |  | (H x OD) |  |
| **First Crush Gen2** |  | Plastic CompleteRX cup | 26 x 70 | c |
| **Ocelco Plastic Pillcrusher** |  | Paper soufflé cup, ¾ ounce | 28 x 37 | a |
| **Rhino Crush** |  | Plastic soufflé cup, ½ ounce | 22 x 37 | b |
| With disposable bag |  |  | (L x W) |  |
| **Metal Handheld** |  | Tablet bag, 2 mil | 66 x 62 | g |
| **MiniTwist** |  | Easy empty bag, 7 mil | 90 x 40 | f |
| **Powdercrush** |  | Powdercrush pouch, 7 mil | 145 x 50 | i |
| **Roc N Crush** |  | Resealable bag, 6 mil | 97 x 64 | h |
| **Quiet Crusher** |  | Crusher bag, 7 mil | 107 x 50 | e |
| **Silent Knight** |  | Silent Knight pouch, 7 mil | 109 x 52 | d |
